# Comparison of chest HRCT severity score in PCR positive and PCR negative clinically suspected COVID-19 Patients

**DOI:** 10.4314/ahs.v21i4.9

**Published:** 2021-12

**Authors:** Hina Hanif Mughal, Syed Muhammad Jawad Zaidi, Hamza Waqar Bhatti, Madiha Maryum, Maria Khaliq, Nasir Khan, Mehwish Kaneez

**Affiliations:** 1 Holy Family Hospital, Department of Radiology; 2 Rawalpindi Medical University, Medicine

**Keywords:** Chest High resolution computed tomography (HRCT), COVID-19, Polymerase Chain Reaction (PCR)

## Abstract

**Background:**

The limitations and false-negative results of Real-time Polymerase chain reaction (RT PCR) in diagnosing COVID-19 infection demand the need for imaging modalities such as chest HRCT to improve the diagnostic accuracy and assess the severity of the infection.

**Objectives:**

The study aimed to compare the chest HRCT severity scores in RT-PCR positive and negative cases of COVID-19.

**Methods:**

This cross-sectional study included 50 clinically suspected COVID-19 patients. Chest HRCT and PCR testing of all 50 patients were done and the chest HRCT severity scores for each lung and bronchopulmonary segments were compared in patients with positive and negative PCR results. Chi-square and Mann Whitney U test were used to assess differences among study variables

**Results:**

Chest HRCT severity score was more in PCR negative patients than in those with PCR positive results. However, the difference was not significant (p=0.11). There was a significant association in severity scores of the anterior basal segment of the left lung (p=0.022) and posterior segment upper lobe of right lung (p=0.035) with PCR results. This association was insignificant for other bronchopulmonary segments (p>0.05).

**Conclusion:**

CR negativity does not rule out infection in clinically suspected COVID-19 patients. The use of chest HRCT helps to determine the extent of lung damage in clinically suspected patients irrespective of PCR results. Guidelines that consider clinical symptoms, chest HRCT severity score and PCR results for a confirmed diagnosis of COVID-19 in suspected patients are needed.

## Introduction

The novel Coronavirus disease 2019 (COVID-19), an emergent outbreak is a distinctive form of progressing viral pneumonia caused by SARS-CoV-2 1. The unique form of viral pneumonia originated from the wet market of Wuhan city of China but has now become a pandemic resulting in a global health crisis 2. The disease varies greatly in intensity ranging from mild flu-like symptoms to severe acute respiratory distress that can ultimately lead to death 3. Due to rapid propagation and booming of cases across the world, it demands timely and error-free diagnosis of patients aiding in the control of the source of infection and disease progression. Currently, the diagnosis of COVID-19 depends upon Real-Time Polymerase Chain Reaction (RT-PCR) along with clinical symptoms and epidemiological history but the false-negative results by PCR are of a big concern to the health-care professionals^4^–^5^.

If a patient is a clinical suspect of COVID-19 infection, (with clinical symptoms, travel, or exposure history), a negative PCR does not necessarily rule out active infection as PCR can have a false negative result 5. It can be due to an inadequate sample containing insufficient cellular material for nucleic acid detection or improper sampling technique 6. Moreover, studies report very low sensitivity of PCR that is between 50–62% 7–8. It also requires trained personnel, expensive laboratory instruments, and is time consuming 8. These factors limit and question the diagnostic ability of RT-PCR in confirming the diagnosis of COVID-19 making it a daunting challenge for the clinicians especially in the developing countries where the health-care resources are scarce. The low sensitivity of PCR can be ameliorated by multiple testing but incorporating this technique will exploit the already sagging health-care system of a developing country.

The High-Resolution Computed tomography (HRCT) scan of the chest is an important imaging technique that aids in the screening, diagnosis, and management of COVID-19. The most common characteristic findings on chest HRCT scan of a COVID-19 pneumonia patient are air space consolidation, traction bronchiectasis, broncho-vascular thickening in the lesion, fibrosis, bilateral, subpleural, or peripheral ground-glass opacities (GGO), and crazy paving appearance (GGO with inter/intralobular septal thickening and each bronchopulmonary segment and lung is scored based on these findings 9–11. However, chest HRCT may be normal in patients with mild symptoms and no pneumonia that limits the role of chest HRCT-scan as a gold standard test for diagnosing SARS-CoV-2 infection 9–10. Nonetheless, a chest HRCT scan is an important investigation in determining the extent and severity of the disease in hospitalized and clinically suspected patients with negative PCR results 5,7,10. The study aimed to compare the chest HRCT severity score in clinically suspected patients of COVID-19 with different PCR results. We also aim to evaluate which regions of the lungs are most commonly affected in clinically suspected patients of COVID-19.

## Methods

A cross-sectional study conducted at the Radiology department of Holy Family Hospital, Rawalpindi, Pakistan which included a total of 50 patients. The clinically suspected patients of COVID-19 pneumonia hospitalized in isolation wards and subsequently tested by PCR were included in the study. The criteria for clinical suspicion were similar to the one used by the National Health Commission, China 10. This criterion involved epidemiological and clinical aspects of the disease including recent travel history, exposure to a confirmed positive COVID-19 case and symptoms including high-grade fever, myalgias, dry cough, shortness of breath, and anosmia. As a negative PCR result does not rule out active infection, all suspected patients were sent for high-resolution Computed tomography (HRCT) scan and the chest HRCT severity scores for each bronchopulmonary segment were calculated separately by two consultant Radiologists.

All scans were performed with Multidetector (16) Toshiba Aquilion CT scanner (Canon Medical Systems Corporation, Ōtawara, Japan) with tube voltage of 120 kvp and current of 350 mA. All patients were scanned in supine position. During the image acquisition, all lung fields from the apices to the bases were covered in a single breath hold and 1mm slice thickness. All images were analyzed for GGO, crazy paving appearance, consolidation, mixed patterns, nodules, and reverse halos sign 11.

Strict infection control measures were adopted during the CT scan of the patients which included strict adherence to PPE guidelines, disinfection of radiology equipment with 70% iso propylalchol and terminal cleaning of the room with diluted bleach, physical and temporal segregation of radiology teams. As per local Hospital policy allotted time was given for suspected or confirmed covid patients in the afternoon after which the room was cleaned and disinfected. After at least 30 minutes the machine was then made ready for re use.

Each bronchopulmonary segment was given a score of 0, 1, or 2 depending upon the above-mentioned parenchymal involvement. The score of 0 indicated negligible parenchymal involvement, the score of 1 showed up to 50% while a score of 2 suggested the involvement of more than 50% of lung parenchyma. The chest HRCT severity score was thus calculated by adding the individual score of each segment 11,12. Findings that were inconsistent with COVID-19 infection were not included in the final chest HRCT severity score.

Patients with known history of Chronic obstructive pulmonary disease (COPD), recent thoracic trauma/surgery, asthma, interstitial lung disease, pulmonary fibrosis, Cor pulmonale, lung malignancies, sarcoidosis, priorly diagnosed with viral pneumonia other than COVID-19 (Influenza, parainfluenza, RSV, etc.), and other conditions that may cause opacities in the lung on chest HRCT were excluded from the study. Patients were clinically assessed for the above mentioned co-morbid conditions. Moreover, we excluded that patients admitted in ICU setting or suffering from severe infection. Furthermore, patients who did not consent to participate in the study were also excluded. The study was ethically approved by the institutional research forum.

The study included all patients fulfilling the inclusion criteria that were admitted in isolation with suspected COVID-19 infection. Due to this our sample size was limited to only 50 patients. Convenience sampling technique was used for patient selection.

The normality of the data was checked using the Shapiro Wilk test. The Median age of the patients was calculated. The overall chest HRCT severity score for each lung and as well as for both lungs were calculated. The difference in median CT severity scores across PCR positive and negative patients was assessed using the Mann Whitney U test. Association between categorical variables was tested using the Chi-square and Fischer exact test. A p-value of less than 0.05 was considered statistically significant. The data analysis was performed on SPSS version 25.

## Results

Out of 50 clinically suspected patients, 32 had positive on PCR while 18 had negative PCR results of SARS-CoV-2. The median age of the patients was 52.5 years. Table 1 delineates the demographics of the study participants with a comparison of median chest HRCT severity scores across PCR positive and negative patients.

**Table 1:** Demographics of the study participants with median (IQR) HRCT severity scores (n=50)

Variable	PCR Positive (n=32)	PCR Negative (n=18)	Total (n=50)	P-value
**Gender**				
Male	22	11	33	0.403*
Female	10	7	17	
**Age**				
Median (IQR)	55(3–72)	49.5(31–78)	52.5(3–78)	0.972**
**Median chest HRCT** **severity Score**				
Right Lung (IQR)	5(1–11)	7.5(0–12)	5.5(0–12)	0.120**
Left Lung (IQR)	5(1–11)	7.5(0–14)	6(0–14)	0.191**
Total Severity score (IQR)	11(0–21)	15.5(1–25)	11.5(0–25)	0.110**

* Chi-square test

** Mann-Whitney U test

Detailed analysis of the chest HRCT severity score of each bronchopulmonary segment revealed that the lower lobe and lingula of the left lung and lower lobe of the right lung had more severity scores than other lobes. Table 2 and 3 show the comparison of severity scores of the bronchopulmonary segments of left and right lung in PCR positive and negative patients.

**Table 2:** Comparison of scores of each lung segment across PCR positive and negative patients

Variable	PCR Positive (n=32)	PCR Negative (n=18)	Total (n=50)	p- value*
	**Left Lung**			
**Anterior segment upper lobe**				
0	23	12	35	0.54
1	8	6	14	
2	1	0	1	
**Apical segment upper lobe**				
0	25	14	39	0.621
1	7	4	11	
2	0	0	0	
**Posterior segment upper lobe**				
0	22	8	30	0.084
1	10	10	20	
2	0	0	0	
**Superior lingular segment**				
0	20	7	37	0.108
1	12	11	23	
2	0	0	0	
**Inferior lingular segment**				
0	13	7	20	0.904
1	19	11	30	
2	0	0	0	
**Superior segment lower lobe**				
0	9	5	14	0.97
1	23	13	36	
2	0	0	0	
**Anterior basal segment lower lobe**				
0	16	5	21	**0.022**
1	16	10	26	
2	0	3	3	
**Medial basal segment lower lobe**				
0	14	4	18	0.073
1	18	12	30	
2	0	0	0	
**Lateral basal segment lower lobe**				
0	10	4	14	0.142
1	22	12	34	
2	0	2	2	
**Posterior basal segment lower lobe**				
0	9	4	13	0.153
1	23	12	35	
2	0	2	2	
0	9	4	13	0.153
1	23	12	35	
2	0	2	2	
	**Right Lung**			
**Anterior segment upper lobe**				
0	23	8	31	0.054
1	9	10	18	
2	0	0	0	
**Apical segment upper lobe**				
0	27	15	42	0.609
1	5	3	8	
2	0	0	0	
**Posterior segment upper lobe**				
0	22	7	29	0.06
1	9	11	20	
2	1	0	1	
**Medial segment Middle Lobe**				
0	22	8	30	0.08
1	10	10	20	
2	0	0	0	
**Lateral segment middle lobe**				
0	18	8	26	0.33
1	14	9	23	
2	0	1	1	
**Superior segment lower lobe**				
0	9	2	11	0.238
1	22	14	36	
2	1	2	3	
**Anterior basal segment lower lobe**				
0	13	4	17	0.07
1	19	12	31	
2	0	2	2	
**Medial basal segment lower lobe**				
0	13	5	18	0.637
1	17	12	29	
2	2	1	3	
**Lateral basal segment lower lobe**				
0	10	4	14	0.77
1	20	13	33	
2	2	1	3	
**Posterior Basal segment lower lobe**				
0	7	6	13	0.244
1	25	11	36	
2	0	1	1	

* Chi-Square test

**Table 2:** Comparison of chest HRCT severity scores across PCR positive and negative patients in left lung

Variable	PCR Positive (n=32)	PCR Negative (n=18)	Total (n=50)	p-value*
**Anterior segment upper lobe**				
0	23	12	35	0.54
1	8	6	14	**0.836**
2	1	0	1	
**Apical segment upper lobe**				
0	25	14	39	0.621
1	7	4	11	
2	0	0	0	
**Posterior segment upper lobe**				
0	22	8	30	0.084
1	10	10	20	
2	0	0	0	
**Superior lingular segment**				
0	20	7	37	0.108
1	12	11	23	
2	0	0	0	
**Inferior lingular segment**				
0	13	7	20	0.904
1	19	11	30	
2	0	0	0	
**Superior segment lower lobe**				
0	9	5	14	0.97 0.623
1	23	13	36	
2	0	0	0	
**Anterior basal segment lower lobe**				
0	16	5	21	**0.022**
1	16	10	26	
2	0	3	3	
**Medial basal segment lower lobe**				
0	14	4	18	0.073
1	18	12	30	0.098
2	0	02	02	
**Lateral basal segment lower lobe**				
0	10	4	14	0.142
1	22	12	34	0.179
2	0	2	2	
**Posterior basal segment lower lobe**				
0	9	4	13	0.153
1	23	12	35	**0.209**
2	0	2	2	

* Chi-square test/Fischer-Exact test

**Table 3:** Comparison of chest HRCT severity scores across PCR positive and negative patients in Right lung

Variable	PCR Positive (n=32)	PCR Negative (n=18)	Total (n=50)	p-value*
**Anterior segment upper lobe**				
0	23	8	31	0.054
1	9	10	19	
2	0	0	0	
**Apical segment upper lobe**				
0	27	15	42	0.609
1	5	3	8	
2	0	0	0	
**Posterior segment upper lobe**				
0	22	7	29	0.06 0.035
1	9	11	20	
2	1	0	1	
**Medial segment Middle Lobe**				
0	22	8	30	0.084
1	10	10	20	
2	0	0	0	
**Lateral segment middle lobe**				
0	18	8	26	0.353
1	14	9	23	
2	0	1	1	
**Superior segment lower lobe**				
0	9	2	11	0.238
1	22	14	36	**0.219**
2	1	2	3	
**Anterior basal segment lower lobe**				
0	13	4	17	0.07 0.080
1	19	12	31	
2	0	2	2	
**Medial basal segment lower lobe**				
0	13	5	18	0.637 0.715
1	17	12	29	
2	2	1	3	
**Lateral basal segment lower lobe**				
0	10	4	14	0.77 0.785
1	20	13	33	
2	2	1	3	
**Posterior Basal segment lower lobe**				
0	7	6	13	0.244 0.297
1	25	11	36	
2	0	1	1	

* Chi-Square test/Fischer-Exact test

## Discussion

The prompt diagnosis of SARS-CoV-2 is important in hospitalized clinically suspected patients to develop appropriate preventive and curative strategies and to also avoid grave complications. In our study, we used a high-resolution CT scan because HRCT helps the radiologists to better visualize lung pathologies than the conventional chest CT scan or X-ray 13. This aided the radiologists to pick up discrete ground-glass opacities in the lung parenchyma. This advantage of HRCT over the conventional chest CT makes it a more diagnostically accurate imaging modality. The results of our study suggest that the detection of abnormalities in chest HRCT aids in confirmation of SARS-CoV-2 infection in clinically suspected patients with false-negative PCR.

In our study, the bronchopulmonary segment of the lower lobes showed higher severity scores than the segments of other regions of the lung. Specifically, the posterior regions of the lower lobes were more involved than other regions. This finding is consistent with a study by Song et al which concluded that regions of lower lobes were predominantly involved in more than 80% of the patients^14^. Yang R. et al reported a similar predominance with the involvement of posterior regions of lower lobes 12. A study on 63 patients by Haseli et al also concluded that consolidation and ground-glass opacities were more common in lower lobes than other regions of the lungs 15. The reason for this particular finding is still unknown but it can help the radiologists to concentrate on the opacities in the lower lobes in assessing a suspected COVID-19 patient.

In our study, the median HRCT severity score of patients with negative PCR results was more (15.5/40) than the patients with positive PCR (11/40). However, this difference was not statistically significant (p=0.11). This finding suggests that PCR negativity does not rule out COVID-19 infection in clinically suspected patients. As a strict and rigorous exclusion criterion was applied in our study, the likelihood of abnormalities in the lungs due to some other pathology was scarce. Moreover, the patients were clinically suspected for SARS-CoV-2 infection which indicates a high probability of false-negative PCR results in such patients as such patients are clinically symptomatic. This finding is consistent with a large-scale study on 1014 patients in China showed high sensitivity of chest CT scan (97%) in diagnosing COVID-19 in highly suspected cases. In this large-scale study, 308 patients with initial negative RT PCR had positive findings consistnt with COVID-19 on CT chest that led to their revaluation^16^. Hence, RT PCR is not independently reliable for diagnosing COVID-19 and more chest HRCT severity scores of clinically suspected patients in our study is suggestive of this conclusion.

A study showed that the sensitivity of RT PCR increases from 79% to 88% if it is combined with CT scan findings^17^. In the context of the above-mentioned findings, the researchers recommend the clinicians to consider PCR results, chest HRCT scan findings, and clinical manifestations of the disease to ultimately diagnose COVID-19. The consideration of all these parameters will aid in prompt diagnosis and treatment of highly suspected patients who may have an initial false-negative result on PCR. Ensuring early diagnosis and prompt treatment of clinically suspected COVID-19 patients by these parameters will aid in reducing the transmission and mortality caused by the virus. Hence appropriate guidelines are needed that combine the use of chest HRCT and PCR for accurate and timely diagnosis of SARS-CoV-2 infection.

Small sample size and having a cross-sectional study design account for a few limitations of our study. Using a control group with proper matching would have made the results of our study more comprehensive. Nonetheless, the results of our study should be given serious consideration as it may help in the early diagnosis of false-negative cases. More studies with large sample sizes are needed to further evaluate the role of chest HRCT scan in diagnosing COVID-19

## Conclusion

The chest HRCT scan is an important and supporting investigation to confirm and rule out COVID-19 infection in clinically suspected patients. HRCT severity score is a reliable index to assess the severity of SARS-CoV-2 infection. The bronchopulmonary segments of posterior regions of lower lobes were more affected than other regions of the lungs. We recommend using the chest HRCT in combination with severity score to improve diagnostic accuracy in clinically suspected or RT-PCR positive COVID-19 patients. There is a need for curation of specific guidelines that consider chest HRCT severity score, clinical manifestations, and PCR results to confirm COVID-9 in clinically suspected individuals.
